# A Local Optima Network View of Real Function Fitness Landscapes

**DOI:** 10.3390/e24050703

**Published:** 2022-05-16

**Authors:** Marco Tomassini

**Affiliations:** Department of Information Systems, University of Lausanne, 1015 Lausanne, Switzerland; marco.tomassini@unil.ch

**Keywords:** global optimization, local optima networks, complex networks, metaheuristics

## Abstract

The local optima network model has proved useful in the past in connection with combinatorial optimization problems. Here we examine its extension to the real continuous function domain. Through a sampling process, the model builds a weighted directed graph which captures the function’s minima basin structure and its interconnection and which can be easily manipulated with the help of complex networks metrics. We show that the model provides a complementary view of function spaces that is easier to analyze and visualize, especially at higher dimensions. In particular, we show that function hardness as represented by algorithm performance is strongly related to several graph properties of the corresponding local optima network, opening the way for a classification of problem difficulty according to the corresponding graph structure and with possible extensions in the design of better metaheuristic approaches.

## 1. Introduction

Search and optimization problems arise in many important fields of science and engineering. Many of these problems are comparatively computationally easy and enjoy efficient algorithmic solutions such as shortest paths and flows in networks, or convex function optimization problems, just to name a few. However, many of the interesting problems do not admit quick and easy solutions. This is the case, for example, of hard combinatorial optimization problems in which the only way to find the globally optimal solution is to essentially enumerate all the admissible solutions and to choose the best one; notable discrete examples among many others being the traveling salesman and the quadratic assignment problems, and the global optimization of an arbitrary real function in the continuous domain. Notable improvements are possible through techniques such as branch-and-bound and similar complete methods or stochastic global optimization approaches, see, e.g., [1], but the task remains difficult. As a consequence, many approximate techniques were developed for both discrete and continuous hard search problems and we shall deal only with this approach in what follows. In general, these methods cannot offer global optimality guarantees or even certified lower bounds on the solution quality but, in exchange, they usually do allow finding good enough solutions in reasonable time. The field of *metaheuristics* [2] developed in this context and comprises several approaches of the latter type: simulated annealing, evolutionary algorithms, and particle swarm optimization are well known examples. In contrast with classical techniques, metaheuristics and similar methods search the space of admissible solutions in ways that try to incrementally improve the current solution or solutions until a predefined stopping condition is met. For this reason, having a good representation of the search space, also called the *fitness landscape*, is a fundamental aspect in metaheuristic search see, for instance, [3]. Thus, fitness landscape analysis has become an important part of metaheuristics because the structure of the fitness landscape of a problem instance is known to be correlated with the performance of a given search algorithm. In this context, a particular representation of a given fitness landscape called *local optima network* (LON) has been proposed recently and its usefulness demonstrated in many studies, see for instance, ref. [4,5] for NK landscapes, ref. [6] for the quadratic assignment problem, ref. [7] for the flowshop scheduling problem, ref. [8] for the minimal autocorrelation binary sequences problem, and [9] for the application of LONs to multi-objective combinatorial optimization problems. The LON is a graph in which the vertices are the local optima of a given problem instance and the edges represent possible transitions between pairs of local optima. This graph thus provides a compressed representation of the instance fitness landscape and can be analyzed in various ways. Once LONs are available for instances of a problem class, the information they provide can be exploited for improving heuristic searches. LONs were inspired by previous work in chemical physics on the structure of search spaces for atomic clusters and of the energy landscape of protein folding [10], where the ensemble of all local optima of the energy function and their transitions were originally called *inherent structures*. Although  until recently the LON approach has mainly be applied in the combinatorial optimization domain, it can also be used in the continuous function optimization field with some modifications. Previous research work in this direction was presented in [11,12,13]. Ref. [11] is the one in which, to our knowledge, basin hopping was first used for sampling the energy surface minima of the RNA folding landscape in order to obtain what they called “basin hopping graph” which is essentially a LON. Ref. [13] is more closely related to the present work but it adopts a different sampling method which leads to the discovery of “funnels” of minima, to be explained later, instead of the full LON. Ref. [12] is particularly relevant for the present work, as we use essentially the same methods and points of view in the LON construction and analysis. However, we extend [12] in several ways, especially in the direction of scaling up the number of space dimensions, in examining the role of edge weights, and in relating complex networks metrics with the empirical hardness of the tested functions.

Following those previous studies, in this paper, we apply the LON idea to the space of continuous real-valued functions. After introducing the LON concept, we describe the sampling methodology, which is different from the one used in discrete fitness landscapes and is based on an optimization heuristic called *basin hopping*, to be explained later. This is followed by a study of the LONs generated from a few well known highly multimodal test functions with the help of complex networks tools and metrics. We then study the relationship between these metrics and the empirical hardness of functions and we extend the analysis to higher dimensions. We conclude with a discussion of the pros and cons of the network approach and with some indications of interesting current and future work.

## 2. Search Spaces and Local Optima Networks

In this section, we summarize the customary LON model for combinatorial optimization problems, followed by a description of the changes needed for dealing with real function global optimization.

### 2.1. Local Optima Networks in Discrete Spaces

Given a discrete problem P and an instance $I_{P}$ of P, $I_{P}$’s *fitness landscape* [14] is a triplet (X,N,f) where X is a set of admissible solutions; $N:X⟶2^{X}$, a function that assigns to every x∈X a set of neighbors N(x), and f:X⟶R gives the fitness or objective value ∀x∈X. The neighborhood N(x) of a solution *x* depends on the “move” operator used to produce a new solution from the current one and on the solution representation. For example, if solutions are expressed as bit strings *x* of length *k*, the mutation of one bit switches a random bit of *x* and so N(x) is the set of the *k* strings at distance one from *x*. Different neighbohoods can be defined depending on the move operator that has been selected.

The LON concept starts from the fitness landscape and compresses it by building a weighted directed graph $G_{w}=\left(V,E\right)$ in which the set of vertices *V* are the *local optima* in the search space and *E* is the set of directed edges joining two vertices (a.k.a. optima) when they are directly connected. Here we assume minimization, maximization is the same with the obvious changes. For a minimization problem a solution x∈X is a local optimum iff $\forall x^{\prime }\in N\left(x\right)$, $f\left(x^{\prime }\right)⩾f\left(x\right)$.

The local minima, i.e., the vertices *V*, are obtained using a best-improvement descent local search in an exhaustive way. Thus, each admissible solution *x* is assigned to a given local optimum, the one at which the local search stops. At the end of the process, the whole search space X becomes partitioned into a number *N* of basins of attraction $B_{i}$, $X=B_{1}\cup B_{2}\cup \ldots \cup B_{N}$, with $B_{i}\cap B_{k}=\emptyset ,\forall i\neq k$.

The edges *E* of the network can be defined in different ways which are almost equivalent, for details see, e.g., [4,8]. In all cases, these directed edges stand for possible transitions between the local optima that they join. The frequency of transition is given by the edge’s weight.

The exhaustive extraction of the problem’s LON is possible for small to medium size problem instances. Larger instances can also be tackled using the sampling methodology of [15].

### 2.2. LON Methodology for Real Function Optimization

We remark that ideas essentially similar to the ones described above for combinatorial spaces are already present in a 2005 paper by M. Locatelli [16], in which he discusses the multilevel structure of global optimization problems in the continuous domain. Now, we discuss the changes needed in the above description to be able to deal with the search spaces of real functions in $R^{n}$. Given a function *f*, the global minimization problem is to find the minimum value *m* of *f* over a feasible domain $X\subseteq R^{n}$ and can be stated as follows:

$$\begin{array}{cc} \min_{x} & \{f(x)\;:\;x\in X\}. \end{array}$$

Usually, one also wants to know the argument, i.e., the point, or set of points, x that provide the minimum value *m* of the function:

$$\begin{array}{cc} \underset{x}{\mathrm{argmin}} & \{x\in X\;:\;f(x)=m\}. \end{array}$$

Here x is a real column vector of scalar variables ${\left[x_{1},x_{2},\ldots ,x_{n}\right]}^{T}$. Maximization is obtained by replacing f(x) with −f(x). The definition also covers constrained optimization problems with a proper definition of X. However, here we will only consider so-called “box constraints” that limit each variable to a segment of the real line, thus restricting the search space to the hyperrectangle X={x:l≤x≤u}, where l and u are vectors of size *n* defining the respective lower and upper bounds on each coordinate of **x**.

In contrast with the discrete case, in topology the neighborhood of a given point p in a real, continuous metric space is defined as an open “ball” of a given radius *r* around p, which is the set of all points in X that are at distance *d* less than *r* from p. Here the standard Euclidean distance is assumed.

In practice, the ball radius will depend on the function of interest and should be chosen appropriately in each case. Having defined the neighborhood, we now need to show how the vertices and edges of the LON $G_{w}=\left(V,E\right)$ are obtained. A method that is capable of finding the minima of a function, as well as the transitions between them called *basin hopping* (BH), is used as a sampling technique.

The outline of the BH algorithm is very simple, see pseudocode Algorithm 1, where solutions s,x,y,z are to be understood as *n*-dimensional vectors.
**Algorithm 1** Basin Hoppings← generate initial solutionx← minimize (f(s))**while** termination condition not met **do**    y← perturb (*x*)    z← minimize (f(y))    x← acceptance (x, z)**end while****return** x,f(x)

The algorithm starts by generating an initial solution *s* either randomly or heuristically. Unless the fitness landscape is flat, this current solution must belong to the basin of attraction of some local optimum whose coordinates *x* are found using a local search procedure starting at *s*. After that the algorithm iterates three stages. First, the current solution *x* is perturbed by some kind of coordinates change yielding the new solution *y*. Next, starting at *y*, a local minimizer finds the new local minimum *z*. There are two possibilities: either *z* is different from *x* or it is the same. In the first case, the algorithm has successfully jumped out of the basin of attraction of *x*. Otherwise, the perturbation has been insufficient and the point *y* belongs to the original basin of attraction, causing the search to find the same minimum again. Finally, the acceptance phase consists of deciding whether the new solution *z* is accepted as the starting point of the next cycle. If it is the same as before the perturbation then it is accepted and the search resumes by trying another perturbation from this point. Otherwise, it is accepted subject to some condition. For example, it could be accepted only if f(z)⩽f(x), or with some probability *p* that decreases with increasing difference |f(z)−f(x)| if f(z)>f(x). The search then continues from the new minimum *z*. The BH heuristic comes from the field of chemical physics and has been successful in finding energy-optimal structures of atomic clusters and simple proteins, see, e.g., [17].

As a sampling technique, to our knowledge BH was first used by Kucharik et al. [11] to characterize the folding landscapes of RNA for building the corresponding LONs which were called *basin hopping graphs*. It was also used in follow-up work on continuous functions by Vinkó and Gelle [12] and by Contreras-Cruz et al. [13]. Our technique for sampling the function minima and their interconnections is built around the BH skeleton but differs from the method of [13] in that a new local optimum is always accepted regardless of its objective function value, since the goal here is to sample a sufficient number of optima besides the best one(s) in order to build a good approximation of the actual LON. The algorithm is outlined in pseudocode Algorithm 2 where s,x,y, and *z* are to be understood as *n*-dimensional vectors.

We now explain the sampling process in more detail. To start with, a real function *f*, a search domain $X\in R^{n}$, a perturbation strength *p*, and a number *q* of sampling points are given. The perturbation strength *p* is important and must be set by trial and error in order to fit the particular function *f* hypersurface. Smaller perturbations are more useful to discover local minima in a highly multimodal function, while larger perturbations are more adapted to slowly varying functions. The number of sampling points *q* depends on the dimensionality of the space *n* but it cannot grow exponentially as the potential number of points to be sampled does. We simply make it proportional to *n*: q=kn with *k* a small integer. Clearly, this will cause the sampling to be less complete as *n* increases but in this way, the task remains feasible. The *q* points can be generated uniformly at random but a better way is to generate them such that they cover the region as well as possible. Here we use quasi-random space-filling sequences obtained according to the Sobol method [18]. This ensures that the initial sampling points are minimally correlated. Next, for each sampling point a **while** loop is executed (line 5) The termination condition of the **while** loop is set as a maximum number of allowed function evaluations. Each time a new minimum is found (We use the standard quasi-Newton method L-BFGS from the Python SciPy library which can also work by numerically approximating the derivatives if they are not available analytically), it is added to the set of graph vertices *V* (lines 4 and 9). Each new directed edge {x,z}, with direction from *x* to *z*, is added to the set of edges *E* (line 10) or, if the edge existed already, its weight is increased (lines 14 and 16) and the search continues from the last minimum found. Notice that self-loops simply count the number of times a perturbation followed by local minimization fell back into the basin of attraction of the minimum from which the perturbation was applied. In continuous spaces, there is a numerical precision issue that is not present in discrete spaces: when the search finds an optimum with the same function value as a previous one (to a certain precision), one must decide whether the optimum has already been found or if it is new (lines 8 and 12). The problem is easily solved by a test on the optima coordinates: the absolute value of their differences must be less than ϵ for all the coordinates for the optima to be the same. We have used ϵ=0.0001. At the end the LON $G_{w}\left(V,E\right)$ is returned. The above sampling procedure differs from the method used in [13]. In the latter, only minima that are monotonously decreasing in objective value, i.e., only improving or equal solutions are accepted. As a consequence, the resulting sampled graphs have long linear branches.
**Algorithm 2** Basin Hopping Sampling**Require:**f(x), $x\in X,X\subseteq R^{n}$, strength *p*, number of sample points *q*1: V=∅,E=∅ //vertices and edges sets2: **for** each sampling point *s* in 1..q **do**3:       x← minimize (f(s))4:       V←V∪{x}5:       **while** termination condition not met **do**6:           y← perturb (x,p)7:           z← minimize (f(y))8:           **if** z∉V **then**9:                V←V∪{x}10:              E←E∪{xz}11:              $w_{xz}\leftarrow 1$12:         **else**13:              **if** z=x **then**14:                  $w_{xx}\leftarrow w_{xx}+1$15:              **else**16:                  $w_{xz}\leftarrow w_{xz}+1$17:              **end if**18:         **end if**19:         x←z20:     **end while**21: **end for**22: return Gw(V,E)

A simple example should be useful to illustrate the above procedure. Let us consider the Branin function B(x,y) in two dimensions which is defined as follows:

$$B\left(x,y\right)={\left(-1.275\;\frac{x^{2}}{\pi^{2}}+5\;\frac{x}{\pi }+y-6\right)}^{2}+\left(10-\frac{5}{4\pi }\right)\cos\left(x\right)+10$$

**Figure 1:**
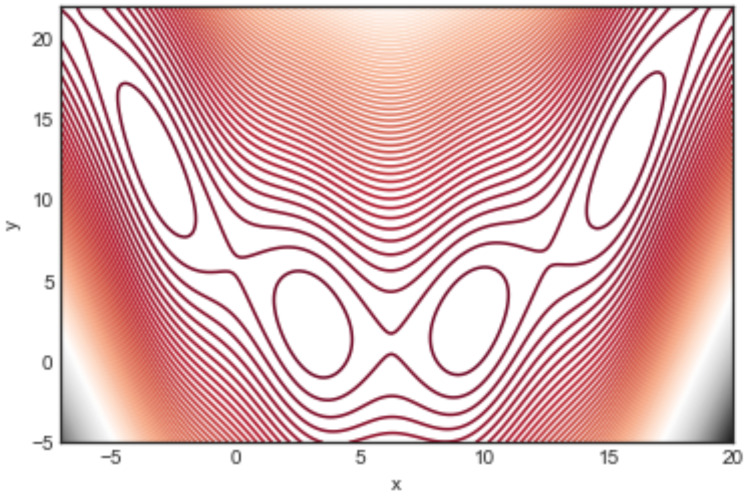
Contour lines representation of the Branin function B(x,y). In the given domain the function has four global minima named 0,1,2,3 from left to right and located at (−π,12.275),(π,2.275),(3π,2.475),(5π,12.875) with B(x,y)≈0.39789.

The function is depicted in Figure 1 by means of contour lines. All of its minima are global with x=π+2πk with *k* a positive or negative integer. In the region [(−7,18), (−5,20)] shown in the figure there are four global minima at: {(−π,12.275), (π,2.275), (3π,2.475), (5π,12.875)} with a function value B(x,y)≈0.39789.

**Figure 2:**
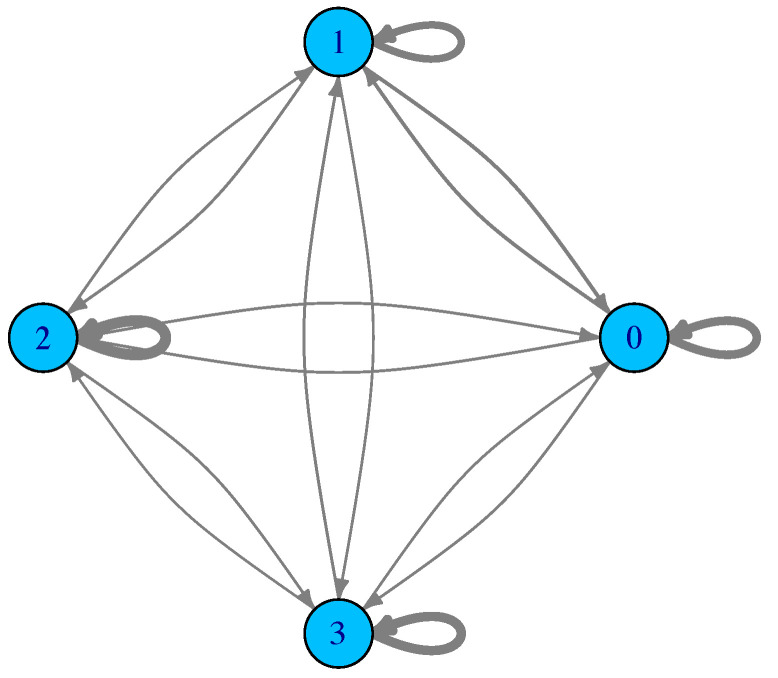
The LON corresponding to the function Branin shown in Figure 1. Vertex labels correspond to the optima and edge thickness is drawn proportional to the corresponding edge weight, which stands for the empirical frequency of transition between the corresponding minima basins.

For such a small number of optima and limited search region, the sampling can be exhaustive, in the sense that all the minima are found and all the possible transitions are recorded too. Figure 2 shows the corresponding LON. Actual edge weights are not shown to avoid cluttering the figure, but the edge thickness is drawn proportional to them. What can be said is that many transitions fall back into the same starting basin and thus the weights of the self-loops are generally higher. Furthermore, by numbering the minima 0,1,2, and 3 from left to right in Figure 1, what the edge weights of the LON show is that transitions to and from the central adjacent minima 1→2 and 2→1 are more frequent than transitions between 0 and 1 and between 2 and 3 in both directions. Transitions between more distant minima, i.e., between 0 and 2, 1 and 3, and 0 and 3, are less frequent, as expected. We end this section with a comment on *neutrality*. Neutral fitness landscapes are those in which there exist large regions in which the objective function has the same value. This notion is particularly important in discrete problems such as the Satisfiability Problem (SAT) and many others. However, most mathematical functions of interest for optimization are either monotone increasing or decreasing, except at special points and thus neutrality is normally not an issue. At the LON level, however, there can be some neutrality. For instance, in the Branin function, above the four degenerate global minima are a neutral set. Neutrality can be easily dealt with in LONs by compressing all connected nodes at the same fitness into a single representative node as explained in [5]. Thus, a compressed LON for the Branin function would consist of a single node. However, doing so would deprive us of the topological knowledge implied by the use of the whole neighborhood of a given optimum which has important implications for search techniques. For this reason, and also because neutrality is usually scarce in LONs derived from real-valued function spaces, we shall not compress neighbors nodes in the sequel and shall represent all of them in the sampled LONs.

## 3. LONS of Some Common Test Functions

The goal of this section is to apply the previous concepts to typical functions belonging to certain classes. When testing new global optimization algorithms it is customary to evaluate them on a given set of contrived functions that are chosen to represent the main kinds of features found in functions arising from general real-life optimization problems. No amount of testing of this kind can prove that one algorithm, say $A_{1}$, is definitely better than another algorithm $A_{2}$ in all possible problems and problem instances. However, such benchmarking is widely used and good results on a sufficiently complete function test set may provide at least some confidence on the expected behavior of an algorithm in general cases. For a detailed presentation of benchmarking methodology and a discussion of its limitations see, e.g., [19].

There exist hundreds of test functions in the literature. Some previous work on synthetic real-valued functions making use of the LON representation appeared in [12,13]. Studying many of them would be too time-consuming but their number can be usefully limited by selecting only a few, provided that they contain the principal structural features that play a role in global optimization. We shall concentrate on some of those functions devoting attention to the relationships between some general properties of function structure and their LON. Among the most important properties, we consider *separability/non-separability*, *unimodality/multimodality*, and their combinations. To start with, unimodal functions give rise to a LON with a single node. There is almost nothing to be learned from such a graph and thus we ignore this type of functions in the sequel. We now consider two typical scalable representative test functions of the highly multimodal type: the Rastrigin function and the Griewank function, whose expressions are given below. The main structural difference is that Griewank is non-separable while Rastrigin is separable. In short, separability means that each coordinate can be optimized independently of the others while this is not possible in non-separable functions in which variables interact. In practice this means that non-separable problems are often harder to solve. We start with the two-dimensional versions of these functions as they are easier to understand and visualize. The effect of scaling up the dimension *n* will be investigated later on.

   Rastrigin function:   

$$R\left(x\right)=10\;n+\sum_{i=1}^{n}\left[x_{i}^{2}-10\;\cos\left(2\pi x_{i}\right)\right]$$

Search region: $x\in {\left[-30,30\right]}^{n}$. Global minimum at $x^{*}=\left(0,\ldots ,0\right)$ and $R(x^{*})=0$.

Griewank function:

$$G\left(x\right)=\sum_{i=1}^{n}\frac{x_{i}^{2}}{4000}-\prod \cos\left(\frac{x_{i}}{\sqrt{i}}\right)+1$$

Search region: $x\in {\left[-60,60\right]}^{n}$. Global minimum at $x^{*}=\left(0,\ldots ,0\right)$ and $G(x^{*})=0$.

**Figure 3:**
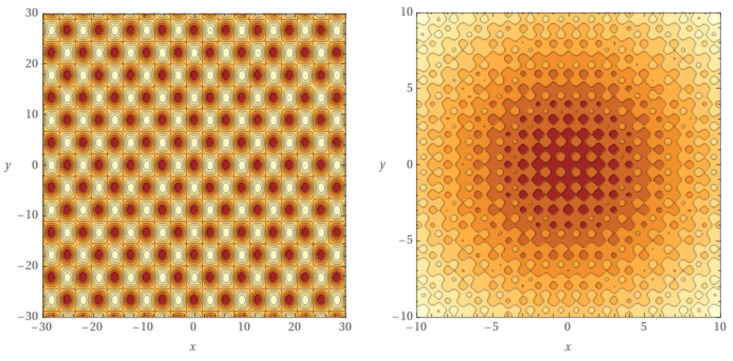
Contour lines plots for two-dimensional Griewank (**left**) and Rastrigin (**right**) functions. Darker areas contain the local minima. Note the different region sizes.

Figure 3 shows the two-dimensional versions of functions R(x) (right image) and G(x) (left image). Both are highly multimodal, with symmetrical equal-valued minima around the global optimum at (0,0). The Rastrigin function has a higher density of local optima (notice the different region sizes).

We shall also use the Schwefel function, defined as follows:

   Schwefel function:   

$$S\left(x\right)=n*418.9829-\sum_{i=1}^{n}x_{i}\sin(\sqrt{\left(\left|x_{i}\right|\right)}$$

**Figure 4:**
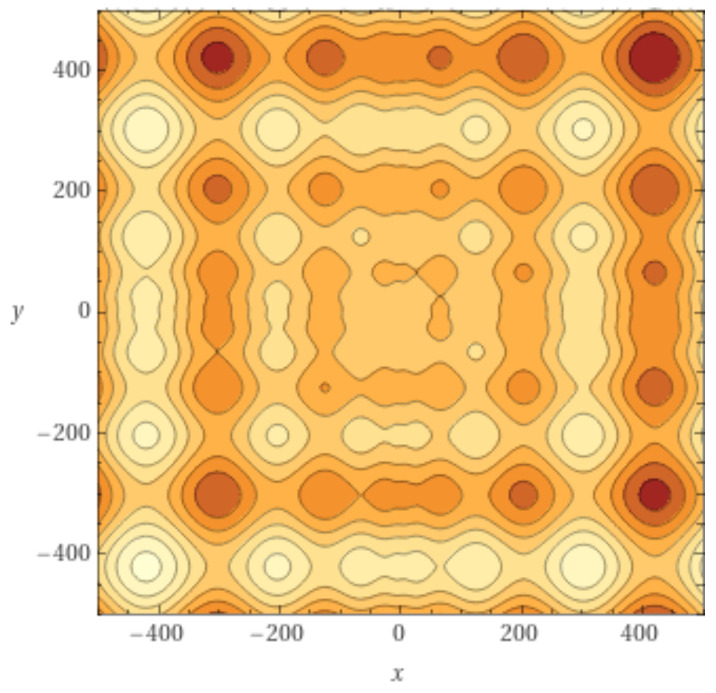
Contour lines representation of the two-dimensional Schwefel function in the domain x∈[−500,500],y∈[−500,500].

Search region: $x\in {\left[-500,500\right]}^{n}$. Global minimum at $x^{*}=\left(420.968746,\ldots ,420.968746\right)$ and $S(x^{*})=0$. The contour lines structure of the two-dimensional *S* function is shown in Figure 4.

### 3.1. Algorithm Performance and Problem Hardness

Some LON metrics are related to the hardness of the function being optimized. It is thus necessary to obtain an empirical evaluation of the optimization hardness of each function, and we do this here for the Griewank and Rastrigin functions. Since there is no agreed upon measure of hardness for arbitrary real functions, we follow common usage and take as a reasonable empirical proxy the success rate over a large number of optimization runs. Due to limited numerical precision, a run is considered successful when the final function value is within 0.0001 of the known global minimum. To reduce algorithm biases, we used two different metaheuristics: differential evolution (DE) [20] and basin hopping (both algorithms were taken from the Python SciPy optimization library using the library standard parameter settings).

We run each algorithm 500 times on each function and record the number of hits and the average number of function evaluations for successful executions. The results are shown in Table 1. We point out that these results, as well as those in Table 2, are well known; they have been replicated here to make them readily available for the sake of the reader. It is clearly seen that, at least for these functions and the choice of algorithms parameters, BH outperforms DE in terms of efficiency (average number of function evaluations), but DE, probably because of its better diversification features due to a population of searchers, is more successful on Griewank. However, here we are mainly interested in the ranking and in both cases Griewank turns out to be the hardest of the two functions.

These results are confirmed when scaling the functions to higher dimensions except in the Griewank case. Table 2 gives the results for dimensions n=5 and n=10. The overall view is that Rastrigin becomes progressively harder with increasing dimension but remains easier than Griewank. The exception is Griewank with n=10 for which the function is hard using DE but becomes easy with BH. This apparent “anomaly” has a straightforward explanation given by Locatelli in [21]. Locatelli found that the Griewank function G(x) becomes easier with increasing dimension using a naive multistart algorithm. In fact, G(x) is the sum of a quadratic convex part and an oscillatory non-convex part and Locatelli showed that, since the convex part minimum is also the global minimum, as *D* increases the gradient of the non-convex part becomes smaller and smaller and the search becomes more and more dominated by the convex minimization, which is easy for algorithms of the Quasi–Newton type such as BFGS [22]. Now, BH is structurally similar to the naive multistart as it uses a BFGS-type routine for local minimization; the only important difference being that start points for local minimization are not randomly chosen in the search space. Instead, they are found by jumping in the neighborhood of the last minimum found. BH thus follows a trajectory through the function minima but is otherwise similar to the multistart approach, which explains its efficiency at higher dimensions. On the other hand, DE is derivative-free and thus it must deal with the exponentially increasing number of local minima without the help of a numerical minimizer as the problem dimension increases.

### 3.2. Local Optima Networks Statistics

The LON methodology is useful mainly for moderately to highly multimodal real functions. Fortunately, these are often the type of functions that are hard to minimize globally and are often found in real-world applications too. Apart from small LONs such as the one corresponding to the Branin function, direct visualization of the graph quickly becomes impractical and of little help as the number of nodes increases beyond a few hundred. Instead, our methodology is based on complex networks concepts and consists of computing several network measures that are known to correlate with problem difficulty and that have been previously found useful in the study of the LONs of problems defined in combinatorial spaces [4,5,6,7]. Based on previous experience, here we use the network metrics briefly defined below for the sake of self-containedness. Fuller explanations can be found in a textbook such as [23].

   Node strength:  

The strength $s_{i}$ is the generalization of the vertex degree to weighted networks and it is defined as:

(1) $$s_{i}=\sum_{j\in N(i)}w_{ij},$$

where $w_{ij}$ is the weight of the edge (i,j) and the sum is over all the neighbors N(i) of a node. In directed graphs, such as those we study here, we have, respectively, the outgoing strength which is computed summing the weights of the outgoing edges from a node, and the incoming strength computed analogously from the incoming edges to a node. Self-loops may be counted either as incoming or outgoing but not both.

   Degree distribution functions:   

The degree distribution P(k) provides, for each degree *k*, the number of nodes with this degree or its frequency. For directed graphs, there are two distinct degree distributions: one for the incoming edges and another for for the outgoing edges. These distributions, and their expected values, are very useful for characterizing the graph as being homogeneous or heterogeneous in terms of the degrees of its vertices.

   Mean shortest paths:   

The average value 〈L〉 of the shortest distances $l_{ij}$ for all the possible pair of vertices i,j in a network is given by:

(2) $$\langle L\rangle =\frac{2}{N(N-1)}\sum_{i=1}^{N}\sum_{j>i}l_{ij},$$

where N=|V| is the number of vertices. Paths are defined only for connected graphs. Since LONs are directed, we require in addition that they be strongly connected. The longest among all the shortest paths is called the *diameter* of the graph.

   Centrality:   

The importance of a vertex in a network can be assessed with the help of *centrality* measures. There are several such measures in common use; here we investigate centrality of optima in a LON network with the use of PageRank.

## 4. Results and Discussion

Below we present first our results of applying the chosen network metrics to the LONs of the Rastrigin and Griewank two-dimensional functions. All computations have been done with custom software, igraph R, and Python NetworkX (All the data used in the analysis will be made available by the author on request ).

### 4.1. Strength

**Figure 5:**
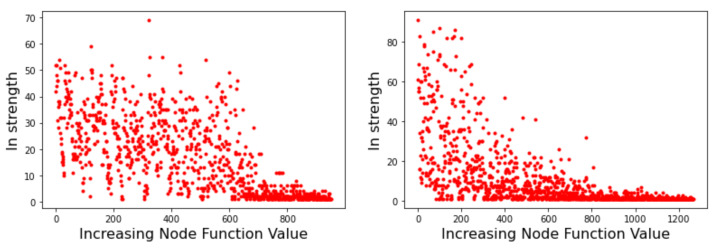
Incoming strength of LON nodes. (**Left image**): Griewank function. (**Right image**): Rastrigin function. Graph nodes are sorted by increasing objective function value.

Node strength is related to the probability of transition between minima basins. As such, it can provide useful information on the likely behavior of optimization methods that search the energy landscape. Figure 5 depicts the strength of the incoming edges to a node for the Griewank LON (left image) and the Rastrigin LON (right image) when the LON nodes are sorted by increasing function value of the corresponding minima to the right of the *x*-axis, i.e., the best minima are near the origin. We see that in the Griewank case there is no apparent relationship between incoming strength and minima fitness. On the other hand, for Rastrigin there is a clear qualitative correlation: nodes corresponding to good minima have high incoming strength. This can be interpreted saying that the best minima are more likely to be reached as they have many more incoming edges from surrounding nodes. Since nodes also represent basins of attraction of the corresponding minima, we can also say that transitions to high-fitness basins are more likely in the Rastrigin case. This is in agreement with the fact stated in Section 3.1 that Griewank is harder to optimize than Rastrigin in two dimensions.

### 4.2. Degree Distribution Functions

**Figure 6:**
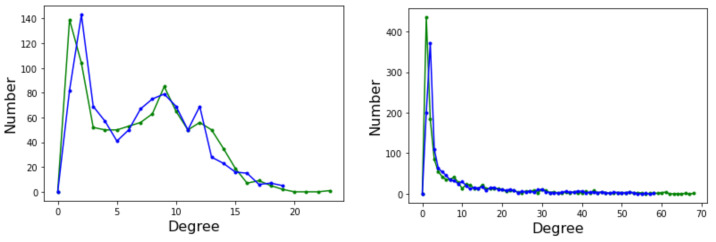
Degree distribution function for incoming (green curve) and outgoing (blue curve) edges. (**Left**): Griewank function; (**Right**): Rastrigin function, both defined in two-dimensional space.

The degree distribution function (DDF) P(k) of a complex network provides the probability that a randomly selected node has degree *k*. P(k) may give some useful indication about the general structure of the network, for example whether it belongs to a known class of distributions such as Poissonian, exponential, or power law. Ignoring the edge weights and self-loops, here we deal with directed graphs and thus we have two distinct degree distributions: the incoming edges distribution and the outgoing edges distribution. Figure 6 depicts the incoming (green curves) and outgoing (blue curves) for Griewank (left image) and Rastrigin (right image) sampled local optima networks.

**Figure 7:**
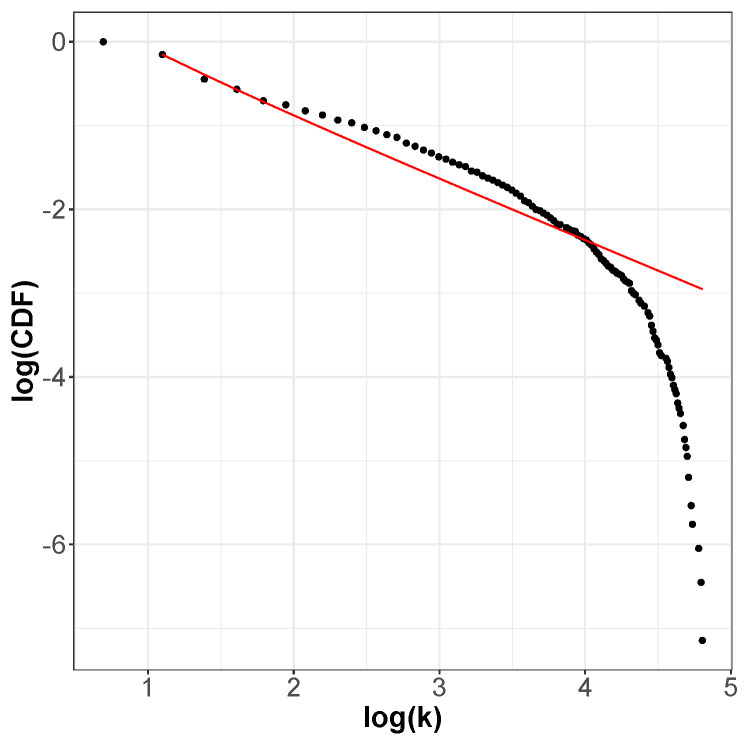
Empirical cumulative degree distribution function of all edges for the Rastrigin LON on double logarithmic scale. The distribution is well fitted by a power law shown by the line in red except near the tail cutoff, as usual for finite and relatively small networks.

The incoming and outgoing distributions are similar in both cases but are very different between functions. Griewank’s LON gives rise to a bi-modal but otherwise rather narrow distribution while the in and out distributions are inhomogeneous featuring longer tails for Rastrigin. Bimodality in the Griewank’s LON is due to the symmetry of the function in which each minimum has four first neighbors, which correspond to the highest peak of the curve and about twelve neighbors at perturbation distance which account for the second smaller one, as can be seen in Figure 4. On the other hand, the long tails in the Rastrigin case indicate that there is a non-negligible number of LON vertices with a large number of incoming and outgoing edges, i.e., the network degree distributions approaches a power law. This is seen by plotting the whole cumulative distribution for incoming and outgoing edges together on double logarithmic scale in Figure 7 with a straight line fit (exponent 1.723, *p*-value = 0.663). This kind of distribution suggests that the high degree nodes could play a role in dynamical processes on LONs and, in fact, we can trace a useful analogy to well known network robustness concepts.

Due to the work of Albert et al. [24], we know that scale-free networks are more resistant than other network topologies to random attacks to their nodes. Even the removal of a sizable fraction of the nodes (and the corresponding edges) does not impact the network function in an essential way up to a point where the network starts to fall apart. Therefore, and the following is to be understood only in a metaphoric sense, not a real one, if we think of a search algorithm as a process that jumps from node to node in the LON, we see that the Rastrigin LON will be more robust in this sense and even if a fraction of the nodes disappear, there will remain other paths to the global optimum. In the Griewank case, on the other hand, the absence of highly connected nodes (hubs) will cause more damage and the search becomes more difficult. In conclusion, there will be more paths of shorter length to the global optimum in the Rastrigin case, making the search easier. This intuition will be confirmed in the next section by inspecting the average path lengths.

### 4.3. Paths and Distances

Again using the metaphor according to which searching the function space translates into walking through the corresponding LON, the statistics characterization of the paths in the latter could be useful to understand the difficulty of the search process. To start with, the average shortest path length (see Section 3.2) is 5.247 for the Griewank LON and 5.905 for the Rastrigin network. Since the number of nodes N=|V| is 951 for the Griewank network and 1267 for Rastrigin, both networks are of the small world type as N=O(logN). Moreover, while the Rastrigin LON has a larger size, it is comparatively smaller, which is in agreement with a well known result on scale-free graphs [23]. The *diameter*, which is the largest among all the shortest paths between pair of nodes, is 29 for the Griewank LON and 34 for the Rastrigin LON. This is the number of hops that a searcher must perform between minima in the worst case, assuming that minima are not visited again once found. Clearly, this is not the case for most optimization metaheuristics in common use and, in addition, the searcher has not enough global knowledge to strictly follow the shortest path. Thus, these values are an interesting indication but cannot be taken too literally.

Another useful network measure is the average length of the shortest paths from all the local optima to the global one for this provides an idea of the difficulty of reaching the global optimum starting anywhere in the search region. Using Dijkstra’s algorithm, for the Rastrigin LON this quantity is equal to 2.93; for the Griewank network it is 3.70. Given that the Rastrigin LON is larger than the Griewank LON, this shows that, on average, a search is likely to reach the global optimum quicker in the Rastrigin search space. This confirms from a different network point of view the fact that Rastrigin is an easier function than Griewank in two dimensions. Of course, this is only a qualitative indication: a search algorithm, being unaware of the structure of the fitness landscape, could do many things other than traveling to the global optimum through a short path, such as cycling among local optima, and thus this information must be taken with a grain of salt. For the sake of clarity, the above measures are grouped in Table 3.

### 4.4. PageRank Centrality

**Figure 8:**
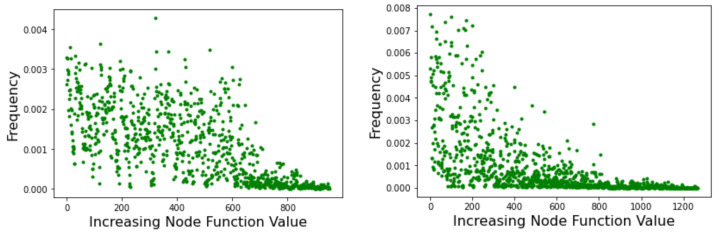
PageRank centrality of all minima versus their objective function values as measured by the frequency of visits during a random walk. Node function values increase to the right of the *x*-axis. (**Left picture**): Griewank network; (**Right picture**): Rastrigin network.

PageRank [25] is an algorithm that provides a measure of the importance of a web page based on how much it is referenced by other important pages. The aim is to capture the prestige of each node in order to rank pages by importance and thus reduce the amount of information to process in a web search. The algorithm can be described as a random walk on the web considered to be a weighted directed graph. In the long time limit, if some conditions are satisfied, the probability distribution reaches an invariant value. This equilibrium probability distribution of the above Markov chain gives the asymptotic frequency of visits to each node of the network. Figure 8 shows the results of running PageRank on the Griewank LON (left image) and the Rastrigin LON (right image). Graph nodes are sorted by increasing objective function value on the x-axis, and the corresponding PageRank centrality, i.e.,  the long-term frequency of visits of each node, is reported on the y-axis. The results fully confirm what was found above by examining the strength of the incoming edges to nodes because the visit frequency depends on the incoming strength. Thus, in the Rastrigin LON, the best minima are also the most central nodes in the network while many nodes in a wide range of fitness have similar centralities in the Griewank network. Therefore, the result in terms of PageRank is fully coherent with the picture that emerged previously and our qualitative argument carries over, indicating that the Griewank function should be more difficult to optimize.

### 4.5. Funnels and Function Difficulty

**Figure 9:**
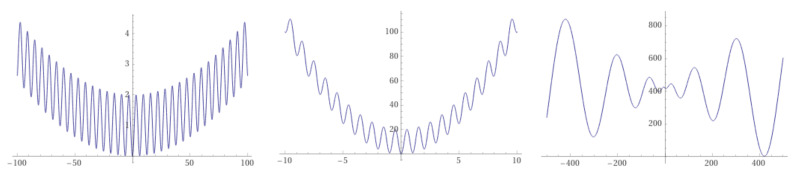
From left to right: one-dimensional Griewank, Rastrigin, and Schwefel functions showing a single-funnel landscape (Griewank, Rastrigin), and a double funnel (Schwefel). Note the different axes scales.

The concept of a *funnel* is a useful one when speaking of the minimization of functions. It was first suggested in chemical physics to help explain the folding of natural proteins into their low-energy state through a collection of convergent pathways in which the energy decreases systematically near the target structure [10,26]. Funnels, understood as a monotonic decreasing sequence of local minima that end up into the funnel bottom, called a sink, have been analogously introduced in LONs of combinatorial spaces by Ochoa and Veerapen [27]. Under the funnel view, fitness landscapes can be classified into single-funnel and multi-funnel. For example, and using one-dimensional functions for clarity, Figure 9 shows (from left to right) that the Griewank and the Rastrigin functions are of the single-funnel type, while the Schwefel function has a double funnel. In general, multi-funnel functions are more difficult to optimize because the search can easily be trapped into a sub-optimal funnel, but the extreme difficulty of minimizing single-funnel energy hypersurfaces for medium to large size proteins [10] suggests that this is not the only source of hardness.

The funnel structure of the LONs of some of the above test functions was studied using non-increasing BH sampling in [13]. The analysis shows that the funnel structure present in the landscapes sketched in Figure 9 carries over to the corresponding LON, which is obvious for these cases but can be useful for real-world functions that have an unknown shape in parameter space. For further details we refer the reader to [13].

**Figure 10:**
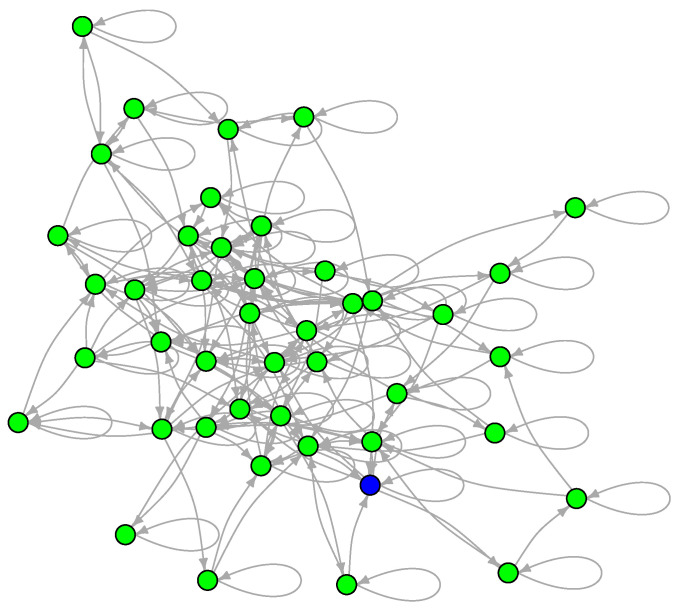
The sampled LON of the Schwefel function in two dimensions. Edge weights are not shown for the sake of clarity. The node corresponding to the global minimum is in blue.

To examine the LON of a multimodal and multifunnel function, we now show the sampled graph of the two-dimensional Schwefel function defined in Section 3 and whose contour lines are shown in Figure 4. This function is interesting for our purposes because its global minimum is far from the origin and because it has a smaller number of minima than either Griewank or Rastrigin. Since there are fewer minima, the LON is small (|V|=44, |E|=183 including self-loops) and can be easily vizualized in Figure 10 where the global minimum is colored blue. Most of the minima in the search region have been sampled but some transitions may be absent. These could be obtained by further increasing the number of BH sampling iterations but the LON as it stands is already highly representative of the function landscape structure.

The mean degree ignoring self-loops and edge directions is 6.32 and the in and out degree distributions are narrow and centered around the mean (we do not plot them because there are too few data for meaningful statistics). As for the typical graph distances, the average path length is 3.16596 and the diameter is 7. Finally, the weighted mean path length to the global optimum from all the other minima is 2.98, i.e., less than three hops, from the point of view of the LON graph topology, remembering that if an edge has weight $w_{ij}$ then its effective length is $1/w_{ij}$ since the $w_{ij}$ represent frequency of transitions between pairs of minima; i.e., remembering that Dijkstra’s algorithm uses weights as lengths, minima connected by an edge with a large weight are effectively “shorter” and thus are more likely to communicate.

Table 4 reports the success rate on the Schwefel function for dimensions two, five, and ten, respectively. Only the figures pertaining to Differential Evolution were reported as BH has troubles converging to the global minimum for this function within the allowed budget of function evaluations.

Comparing Schwefel in two dimensions with the corresponding results using DE for Griewank and Rastrigin (see Table 1), it is apparent that Schwefel is far easier than Griewank and approximately as difficult as Rastrigin, despite the higher number of local minima of the latter. We see thus that Schwefel’s multifunnel is more than compensated for by the non-separability and high multimodality of the single-funnel Griewank. In conclusion, multi-funnels surely make the search harder but there are several other factors influencing optimization difficulty such as high multimodality, non-separability, deception and conditioning, some of them being of numerical nature rather that topological. This is why modern benchmark test suites try to incorporate all of these features in order to duplicate the typical difficulties that are believed to occur in continuous domain search in practice. A good example of this approach is ref. [28].

### 4.6. Scaling to Higher Dimension

In this section, we investigate how and when the indications drawn in the two-dimensional case extend to higher dimensions. First of all, it is useful to obtain an understanding of how the number of local minima scales with dimension for the multimodal functions used in the text. To this end, a reasonable estimate, which is a lower bound, can be obtained with a simple multistart algorithm as described in pseudocode Algorithm 3. The algorithm generates a large number *q* of random starting points and then, for each of them, locally minimizes the function.
**Algorithm 3** Multistart Minima Sampling**Require:**f(.), bounding box $B=x\in {\left[a,b\right]}^{n}$, number of starting points *q*  create empty list of minima *M*  **for** i←1 to *q* **do**       s← generate a random solution in *B*       m← minimize (*f*(*s*))       **if** m∉M and *m* not out of bounds *B* **then**          add *m* and f(m) to *M*       **end if**  **end for**  **return** *M*

The results obtained using this technique are summarized in Table 5, where the number of local optima is reported for dimensions n=2,5, and 10 for each function. The hyperboxes considered for each function are $x\in {\left[-60,60\right]}^{n}$ for the Griewank function, $x\in {\left[-30,30\right]}^{n}$ for Rastrigin, and $x\in {\left[-500,500\right]}^{n}$ for the Schwefel function. Except for the case n=2 for which the figures are close to the actual values, these numbers are lower bounds and thus the true number of optima is surely underestimated.

The values in Table 5 are a manifestation of what is usually called the “curse of dimensionality”, meaning the rapid increase of problem volume associated with increasing space dimension. For the highly multimodal functions, we here consider the number of minima consequently grows rapidly, making sampling, and also global optimization, more difficult and time-consuming for higher dimensions. We mention in passing that this is not true for the Griewank function in which the number of minima first increases but then decreases for n>5 for the reasons sketched in Section 3.1. For the Schwefel function going to n=5 is not a problem but for Griewank and especially Rastrigin the process, although it can technically be performed, is very lengthy if good sampling is required.

**Figure 11:**
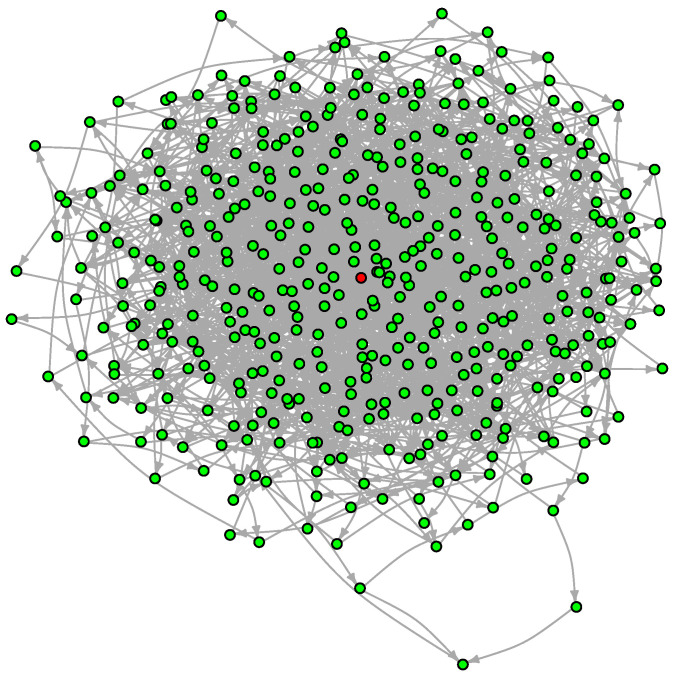
The LON of the Griewank function in five dimensions in the box ${\left[-10,\;10\right]}^{5}$. Self-loops, edge weights and node labels are not shown for the sake of clarity. The node corresponding to the global minimum is in red.

For the sake of illustration, we will take the Griewank function at n=5 in the reduced box $x\in {\left[-10,\;10\right]}^{5}$ for which Algorithm 3 finds about 500 local minima. The resulting LON is shown in Figure 11.

**Figure 12:**
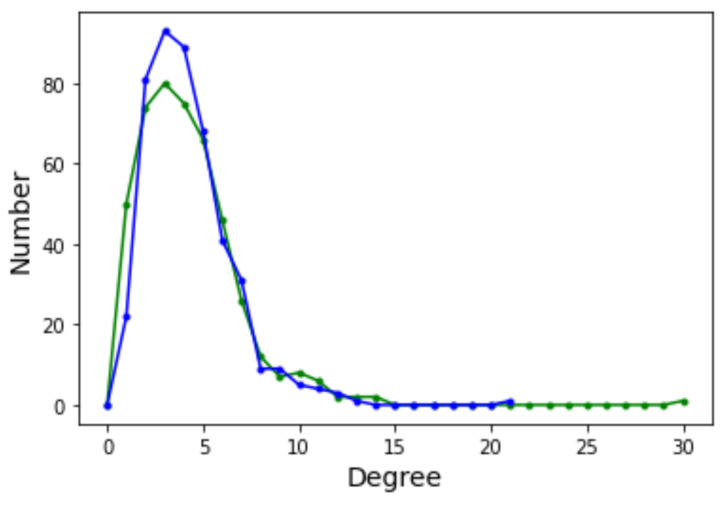
Degree distribution functions for the Griewank LON in five dimensions. Green curve: incoming edges; blue curve: outgoing edges.

The graph has 457 vertices and 1939 edges without considering self-loops for a mean degree of 8.49. The degree distribution function is shown in Figure 12. With respect to the DDF for the case n=2 (see Figure 6 in Section 4.2) we observe that the shape of the curves are intermediate between Griewank and Rastrigin in two dimensions. In the present case with n=5 the curves have a longer tail to the right but not as extended as for Rastrigin. This suggests that there are more nodes with relatively high in and out degrees and we saw in Section 4.2 that this is an indication that searching the optima becomes easier. Indeed, the Griewank function in five dimensions is less hard than the same function in two dimensions according to Table 1 and Table 2 in Section 3.1.

**Figure 13:**
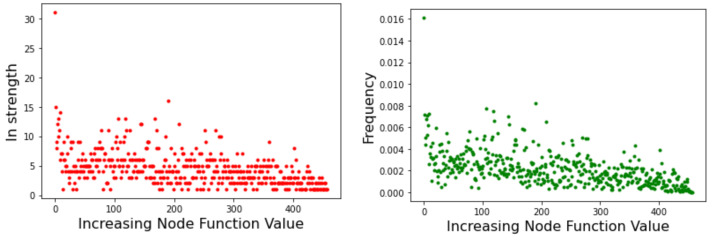
Griewank function at dimension n=5. Nodes are sorted by increasing objective function value. (**Left image**): incoming strength. (**Right image**): node centrality according to the Page Rank algorithm.

The above indications are confirmed by the incoming edges strength and by the related Page Rank centrality depicted in Figure 13 with lower function values to the left. Here we see that high-fitness nodes tend to have, on average, a higher incoming strength and, correspondingly, high centrality. Indeed, the global optimum is the node with both highest strength and highest centrality.

The average path length is 4.28, logarithmic in |V|, but the region of interest, i.e., $x\in {\left[-10,10\right]}^{5}$, is smaller than the one considered at n=2 which was $x\in {\left[-60,60\right]}^{2}$. In fact, the shrinking of the box is approximately compensated by the increase in volume at higher dimension.

A benefit of the LON representation can be seen here. A problem that would be very difficult or even impossible to describe in the original five-dimensional metric space is transformed into a graph in which neighbor and other graph relationships corresponding to features in the original problem space can be easily computed, and sometimes even visualized. This is an advantage because in high dimensions, the concept of closeness becomes unclear.

**Figure 14:**
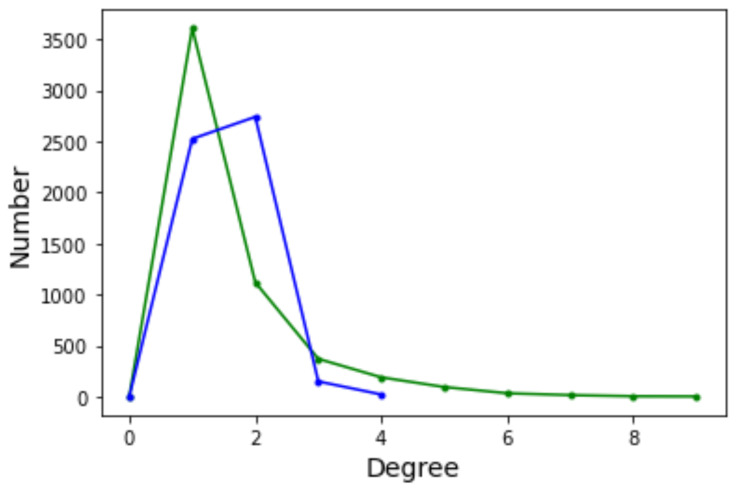
Degree distribution functions for the Schwefel LON in five dimensions. Green curve: incoming edges; blue curve: outgoing edges.

As a final example, let us consider the LON corresponding to the Schwefel function in the domain $x\in {\left[-500,\;500\right]}^{5}$. This LON is much larger with |V|=5436 and |E|=8536 excluding self-loops, which is clearly too large for direct visualization to make sense. In this case we know from Table 5 that there are about 11,000 local minima. The sampling process finds half of them in reasonable time but the number of directed edges, i.e., the transitions between minima, is certainly underestimated with a mean degree of only 3.14 not counting self-loops. This makes computing path lengths less reliable and so we do not do it. The DDF is also influenced by the undersampling of graph edges of course, but it is narrow and concentrated around the mean anyway, see Figure 14. However, the incoming edge distribution (green curve) has a longer tail, which is an indication of the presence of some nodes with a high incoming degree. As we saw in Section 4, this suggests that those nodes are easier to reach in a search process.

**Figure 15:**
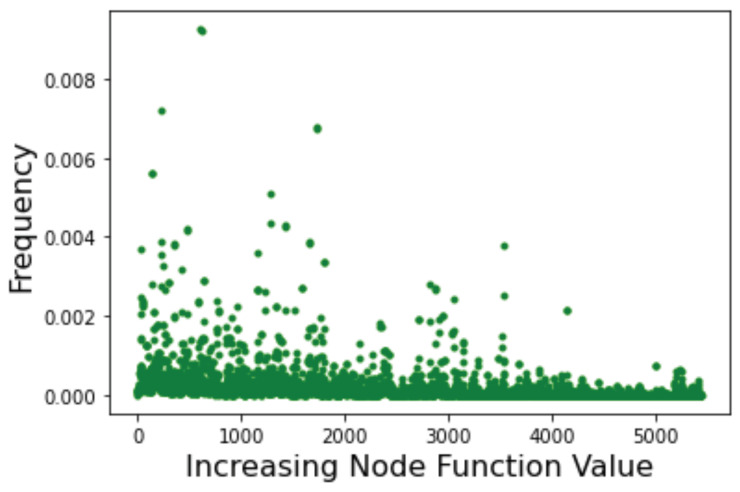
The Page Rank centrality of nodes in the Schwefel LON. Nodes represent function minima and are ordered by increasing objective function value.

Below we also give the Page Rank centrality results in Figure 15. This scatter plot provides qualitative confirmation that, indeed, the most central nodes are among those corresponding to low values of the objective function of the corresponding minima. In other words, good minima should be relatively easy to locate during a search. This is in agreement with the fact that Schwefel should remain “easy” in higher dimensions as shown in Table 4.

## 5. Conclusions

In this work, we applied the LON model, which abstracts the global fitness landscape of a continuous function in some domain of multidimensional real space into a weighted directed graph. The approach was applied previously with success to discrete spaces of the combinatorial optimization type. Starting with simple two-dimensional functions chosen among multimodal standard test functions, we showed that the methodology for sampling the given domain can be based on basin hopping, a trajectory method that jumps from a local minimum to another neighboring local minimum. The LON graph thus obtained can be examined using several network metrics that, altogether, qualitatively correlate with the hardness of the corresponding function, as empirically measured by success rate using some metaheuristic for global optimization. The method scales to higher dimensions to a certain extent but, as all computations on quickly increasing search volumes, it suffers from the “curse of dimensionality” phenomenon. Although sampling could be performed in principle for dimensions equal or larger than ten, in practice it takes too long and the graphs obtained thus are too sparse to be a good representation of the original real space landscape. This is especially the case for highly multimodal functions, which are the more frequently adapted and more interesting for the LON representation, but whose number of minima grows too quickly with increasing dimensions. Clearly, the limitation is less stringent for other slowly varying functions. Nevertheless, we showed on five-dimensional versions of the functions that the method has the merit of being able to represent relationships between function minima and the transitions between them in a way that would not be possible in the original multidimensional metric space on which these functions are defined. Although the present work is just a first step, examining the LON of a given function or function class in various ways could in principle provide useful general information for optimization methods of the metaheuristic type, as these approximate algorithms make heavy use of the underlying search space structure. This aspect is left for future work but see Homolya and Vinko [29] for a recent study in which LONs are used in the context of Memetic Differential Evolution. Furthermore, we used difficult but contrived functions that are typically found in standard benchmark test suites. However, standard test functions are not necessarily representative of functions arising in applications. It would thus be interesting to test the methodology on real-world continuous optimization problems in the future. A first step in this direction was made in [13] and applications to simple machine learning problems have been shown using a related approach in [30].

## Figures and Tables

**Table 1:** Fraction of optimization runs that found the global minimum for the Griewank and Rastrigin functions in two dimensions for DE (left column) and BH (right column). The average is over 500 optimization runs in each case. A budget of $3.0\times 10^{3}$ function evaluations was allowed per run and the search region is ${\left[-30,\;30\right]}^{2}$. The mean number of function evaluations when the global minimum has been found is in parentheses.

Success Rates		
	DE	BH
Griewank	0.464(2479)	0.050(1528)
Rastrigin	0.930(2178)	0.980(928)

**Table 2:** The fraction of optimization runs that found the global minimum for the Griewank and Rastrigin functions for dimension n=5 (left half of the table) and n=10 (right half) using DE and BH. The averages are over 500 optimization runs in each case. A budget of $3.0\times 10^{4}$ function evaluations has been allocated per run and the search region are, respectively, ${\left[-30,30\right]}^{5}$ and ${\left[-30,30\right]}^{10}$. The mean number of function evaluations when the global minimum has been found is in parentheses.

Success Rates				
	*n* = 5		*n* = 10	
	DE	BH	DE	BH
Griewank	0.157 (25,496)	0.080 (18,575)	0.016 (96,573)	0.926 (75,314)
Rastrigin	0.818 (14,754)	0.352 (14,945)	0.496 (105,202)	0.165 (107,571)

**Table 3:** Typical distances in the Griewank network (top line) and the Rastrigin network (bottom line). First column: diameter (largest among all shortest paths). Second column: average shortest path computed according to Equation (2) in Section 3.2. Third column: average path length to the global optimum from all other minima.

	Diameter	Av. Shortest Path	Av. Path to GO
Griewank	26.423	5.247	3.70
Rastrigin	33.333	5.905	2.93

**Table 4:** Fraction of optimization runs that found the global minimum for the Schwefel function using DE for dimensions n=2,5,10. The averages are over 500 optimization runs in each case. The search region is ${\left[-500,\;500\right]}^{n}$. The allocated budget of function evaluations is $3.0\times 10^{3},10.0\times 10^{3}$, and $30.0\times 10^{3}$ for n=2,5 and 10, respectively. The mean number of function evaluations when the global minimum has been found is in parentheses.

Success Rates			
Schwefel	*n* = 2	*n* = 5	*n* = 10
	0.910 (1115)	0.866 (6265)	0.675 (26,992)

**Table 5:** Estimated number of local minima for Schwefel, Rastrigin, and Griewank functions for dimensions n=2,5,10 defined in $x\in {\left[-500,500\right]}^{n}$, $x\in {\left[-30,30\right]}^{n}$, and $x\in {\left[-60,60\right]}^{n}$, respectively.

Number of Local Minima			
	*n* = 2	*n* = 5	*n* = 10
Schwefel	∼50	∼11,000	>85,000
Rastrigin	∼3600	>100,000	>100,000
Griewank	∼528	>95,000	>6000

## Data Availability

Not applicable.
